# Experimental Study of the Potential Role of *Salmonella enterica* subsp. *diarizonae* in the Diarrhoeic Syndrome of Lambs

**DOI:** 10.3390/pathogens10020113

**Published:** 2021-01-23

**Authors:** Dimitris C. Chatzopoulos, Natalia G. C. Vasileiou, Katerina S. Ioannidi, Angeliki I. Katsafadou, Vasia S. Mavrogianni, Charalambia K. Michael, Eleni I. Katsarou, Emmanouil Karavanis, Nikolaos Papadopoulos, Afroditi Sbiraki, Labrini V. Athanasiou, Charalambos Billinis, George C. Fthenakis

**Affiliations:** 1 Veterinary Faculty, University of Thessaly, 43100 Karditsa, Greece; vetdchatzop@gmail.com (D.C.C.); kate_ioan@windowslive.com (K.S.I.); agkatsaf@vet.uth.gr (A.I.K.); vmavrog@vet.uth.gr (V.S.M.); cmichail@vet.uth.gr (C.K.M.); elekatsarou@vet.uth.gr (E.I.K.); lathan@vet.uth.gr (L.V.A.); billinis@vet.uth.gr (C.B.); 2 Faculty of Animal Science, University of Thessaly, 41110 Larissa, Greece; vasileiounat@gmail.com; 3 Histopathology Laboratory, 3rd Veterinary Hospital of Greek Army, 57000 Thessaloniki, Greece; ekaravanis@gmail.com (E.K.); npapadovet@yahoo.gr (N.P.); 4 Veterinary Laboratory of Halkida, Hellenic State Veterinary Service, 34150 Halkida, Greece; asbiraki@gmail.com

**Keywords:** diarrhoea, diarrhoeic syndrome, ewe, intestine, lamb, liver, mastitis, milk, *Salmonella*, sheep

## Abstract

The objectives of this experimental work were the evaluation of the potential role of *Salmonella enterica* subsp. *diarizonae* in diarrhoeic syndrome in lambs and the investigation of facets of the pathogenesis of the infection. In total, 12 lambs were challenged orally on the first day of life, with a *S. enterica* subsp. *diarizonae* isolate from a clinical case of diarrhoeic syndrome. Sequential blood, faecal and buccal samples were collected from lambs and faecal and milk samples were taken from their dams. Lambs were euthanised 1, 2, 4, 7, 10, 14 and 21 days after challenge. Samples were processed for recovery of the challenge organism; they were also subjected to examination by PCR for detection of the *invA* gene. Tissue samples from lambs were also examined as above and histopathologically. *S. enterica* subsp. *diarizonae* was recovered from faecal samples of all lambs, in total, from 45/77 samples (median duration: 2.4 days post-inoculation). It was also recovered from buccal samples (10/77) from seven lambs (median duration: 0.8 days), and from tissue samples (small intestine, abomasum, liver, gallbladder) of nine lambs. It was recovered from two consecutive milk samples from the same ewe, but not from any faecal sample from ewes. The *invA* gene was detected in samples from all lambs (median duration: 5.5 days in faecal and 1.3 days in buccal samples), as well as in milk samples from three ewes. Histopathological findings included abomasitis with subepithelial presence of eosinophils, lymphocytes and plasma cells, consistently observed in all lambs. In the small intestine, salient lesions initially included distension and oedema of intestinal villi, leucocytic infiltration and hyperplasia of lymphoid nodules with apparent germinal centres; this was followed at later stages by atrophy and/or degeneration of the lymphoid tissue of the intestine with marked subepithelial infiltration of lymphocytes, plasma cells and eosinophils.

## 1. Introduction

The genus *Salmonella* includes two species: *Salmonella bongori* and *Salmonella enterica*. The latter includes six subspecies: *S. enterica* subsp. *enterica* (I), *S. enterica* subsp. *salamae* (II), *S. enterica* subsp. *arizonae* (IIIa), *S. enterica* subsp. *diarizonae* (IIIb), *S. enterica* subsp. *houtenae* (IV) and *S. enterica* subsp. *indica* (V). Isolates currently classified in *S. enterica* subsp. *diarizonae* were first detected in faecal samples from reptiles [1] and initially included into the *Salmonella* “Arizona” group, later termed subgenus Arizona or subgenus III. The subgenus was subsequently divided into *S. enterica* subsp. *arizonae* (IIIa) and *S. enterica* subsp. *diarizonae* (IIIb), based on differing reactions in biochemical tests and genomic relatedness [2].

At least 336 distinct serovars of *S. enterica* subsp. *diarizonae* have been detected, which was approximately 13% of all recorded serovars in the *S. enterica* species [3,4]. *S. enterica* subsp. *diarizonae* isolates have been most frequently recovered from samples collected from cold-blooded animals [5,6] or the environment and may also be harboured by domestic (e.g., sheep, details below) or wild [7,8] warm-blooded animals, as well as humans. *S. enterica* subsp. *diarizonae* mainly colonises the gastrointestinal tract of hosts, specifically the anterior part of the small intestine.

*S. enterica* subsp. *diarizonae* is considered as the most frequently detected *Salmonella* subspecies in sheep, but it has not been studied widely in the literature. Most *S. enterica* subsp. *diarizonae* infections are caused by isolates with antigenic type 61:k:1,5,(7) [9], although serovars with minor modifications or with incomplete antigenic structure have also been detected.

The organism was isolated from samples from sheep in the United Kingdom [10], Norway [11,12] and Germany [13], with isolation rates varying from 1% to 76% of samples. Further, during abattoir studies in the United Kingdom [14], Switzerland [15] and Sweden [16], isolation rates were always less than 1% of sheep carcasses.

The organism is a cause of clinical diseases in sheep [17]. It has been associated mainly with gastrointestinal disorders in lambs [18,19,20], abortion in ewes [21] and chronic proliferative rhinitis in adult sheep [22,23].

Recovery of the organism in samples from lambs with diarrhoea can potentially lead to diagnostic problems regarding the causal agent(s) of the problem. We have already described an outbreak of diarrhoeic syndrome in a flock of sheep, in which *S. enterica* subsp. *diarizonae* was isolated from faecal samples from a lamb with clinical signs, as well as from samples from a clinically healthy ewe in the same farm [20]. Despite previous relevant studies, the possible role of the pathogen in the aetiology of the diarrhoeic syndrome in lambs has not been fully clarified.

The objectives of this experimental study were the evaluation of the potential role of *S. enterica* subsp. *diarizonae* in the diarrhoeic syndrome in lambs and the investigation of facets of the pathogenesis of the infection.

## 2. Results

### 2.1. Clinical Findings

All lambs in the study were clinically healthy before challenge. Only one of the inoculated lambs developed clinical signs within 12 h after challenge (incidence rate: 0.083, 95% confidence interval: 0.015–0.354); the clinical signs lasted until D2 and included increased rectal temperature (>42.0 °C), diarrhoea (watery and yellow-coloured), dullness and depression, recumbency, and increased respiratory rate (>55 min^−1^). The control lambs remained healthy throughout the study (*p* = 0.75 for presence of clinical findings between inoculated and control lambs).

No ewe showed any clinically evident abnormalities during the study.

### 2.2. Haematological Findings

The only significant difference was seen in lymphocyte numbers after D4, which increased as the study progressed and were higher in inoculated lambs (*p* < 0.042) (Table 1). Nevertheless, even in these animals, the findings were within the proposed respective reference range (reference taken into account as presented by Roger [24]). No other differences were seen in haematological values between inoculated lambs and controls; no morphological abnormalities were detected in leucocytes. Details are shown in Table 1.

### 2.3. Bacteriological Findings

#### 2.3.1. Faecal Swab Samples from Lambs

*Salmonella* was not isolated from any faecal swab sample from any lamb in the study, before challenge. After inoculation, the challenge organism was isolated from faecal samples of all lambs (1.000) at least once; intermittent bacterial isolation was recorded in three lambs. In total, the organism was isolated from 45 of 77 (0.584) samples collected from the lambs post-inoculation. Median time of first bacterial isolation was 6 h post-inoculation and median duration of bacterial isolation was 2.4 days. Details are presented in Table 2.

The organism was not isolated from any faecal swab sample from the uninfected control lambs.

#### 2.3.2. Buccal Swab Samples from Lambs

*Salmonella* was not isolated from any buccal swab sample from any lamb in the study, before challenge. After inoculation, the challenge organism was isolated from buccal samples from seven lambs (0.583) at least once. In total, the organism was isolated from 10 of 77 (0.130) samples collected from the lambs post-inoculation. Median time of first bacterial isolation was 1 d post-inoculation and median duration of bacterial isolation was 0.8 days. Details are in shown Table 2.

The organism was not isolated from any buccal swab sample from the uninfected control lambs.

#### 2.3.3. Faecal Swab Samples from Ewes

*Salmonella* was not isolated from any faecal swab sample from any dam of the inoculated lambs, before or after inoculation of these lambs. Further, the organism was not isolated from any sample from the dams of the uninfected control lambs.

#### 2.3.4. Milk Samples from Ewes

*Salmonella* was not isolated from any milk sample from any dam of the inoculated lambs before the inoculation of these lambs. Thereafter, *Salmonella* was isolated from two milk samples, collected from the same ewe, which was the dam of an inoculated lamb, on two consecutive sampling occasions (D4, D7). From the buccal samples of the lamb of that ewe, *Salmonella* was also consistently isolated from D2 to D7.

The organism was not isolated from any milk sample from the dams of the uninfected control lambs.

#### 2.3.5. Tissue Samples from Lambs

*Salmonella* was isolated from tissue samples of nine lambs (0.750). In total, the organism was isolated from 15 of 48 (0.313) tissue samples collected from the euthanised lambs post-inoculation. Specifically, it was isolated from the small intestine of six lambs (0.500), the abomasum of four lambs (0.333), the liver of three lambs (0.250) and the gallbladder of two lambs (0.167) (Table 3). There was no association between the concurrent isolation of *Salmonella* from tissue samples and the recovery from faecal samples from the same lamb on the day of euthanasia (*p* > 0.22) (Table 4).

The organism was not isolated from any tissue sample from the uninfected control lambs.

#### 2.3.6. Identification of Isolates Recovered and Serological Typing

Subsequent detailed identification of the isolates recovered from the experimental animals or their dams, confirmed their identity as *S. enterica* subsp. *diarizonae*. All eight isolates in which serotyping was performed, were confirmed as *S. enterica* subsp. *diarizonae* serotype 61:k:1,5,(7).

### 2.4. Molecular Findings

Each sample that yielded the expected PCR product was interpreted to have harboured *S. enterica* subsp. *diarizonae*. Detailed results of detection of the *invA* gene of *Salmonella* spp. in samples from inoculated lambs or their dams are given in Table 5, Table 6 and Table 7. In total, the *invA* gene was detected in samples from all inoculated lambs and from samples of two of their dams. In all samples (independent of type) that had yielded *Salmonella* at the microbiological examination, the *invA* gene was subsequently detected (1.000).

Median duration of detection of the gene was 5.5 days (0.38–17.5) in faecal samples from lambs, 1.3 days (0–4.8) in buccal samples from lambs and 0 days (0–5.5) in milk samples from ewes.

The gene was not detected in any sample from the uninfected control lambs.

### 2.5. Pathological Findings

#### 2.5.1. Gross-Pathological Findings

During post-mortem examination, there was swelling of the abomasal and the intestinal wall; the latter was also turgid. In some cases, there was fibrinohaemorrhagic enteritis. Lesions were located more prominently at the ileum, especially in lambs euthanised up to D7; in lambs euthanised afterwards, there were lesions also in the jejunum and colon; in all cases, no macroscopic lesions were seen in the duodenum. The mesenteric lymph nodes were enlarged.

#### 2.5.2. Histopathological findings

**Figure 1:**
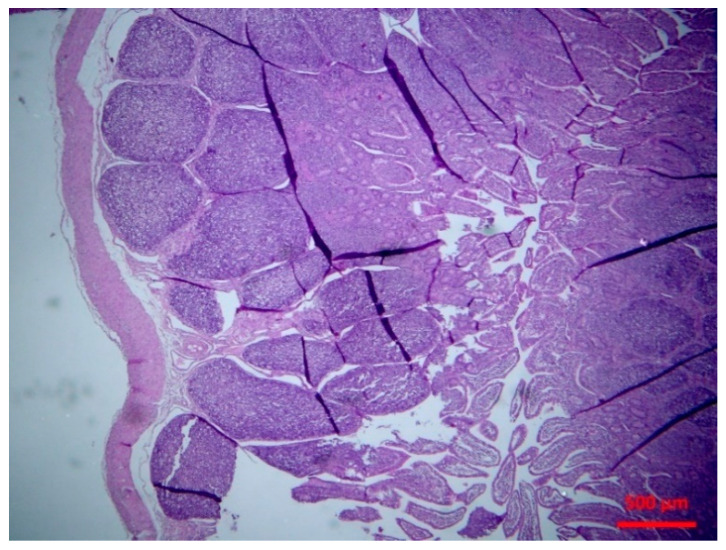
Small intestine: normal lymphofollicular tissue (haematoxylin and eosin (H&E), bar 500 μm).

**Figure 2:**
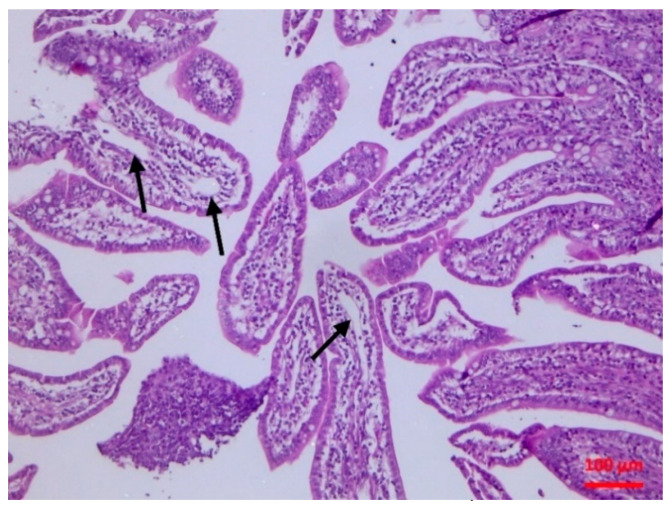
Small intestine mucosa: dilatation of lymphatic vessels (arrows) (H&E, bar 100 μm).

**Figure 3:**
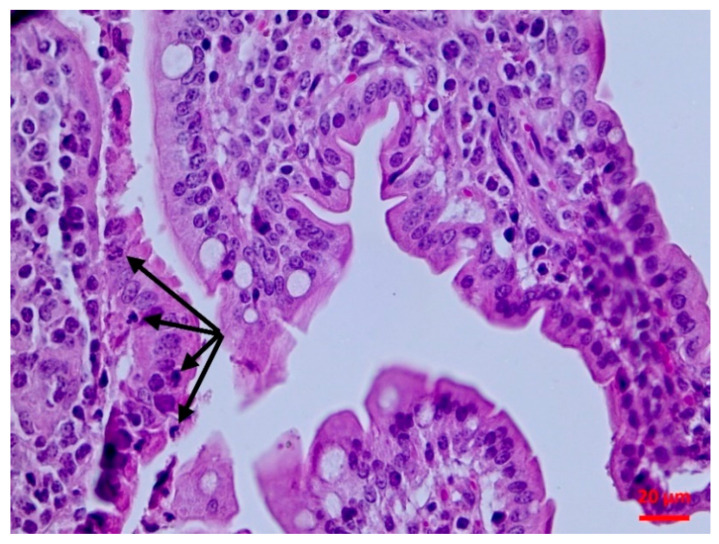
Small intestine mucosa: neutrophilic infiltration (arrows) (H&E, bar 20 μm).

**Figure 4:**
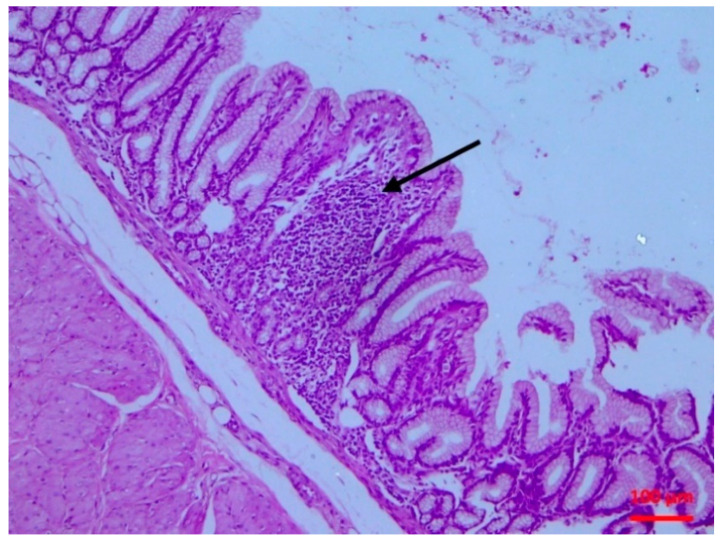
Small intestine mucosa: lymphocytic infiltration of the villous lamina propria (arrow) (H&E, bar 100 μm).

**Figure 5:**
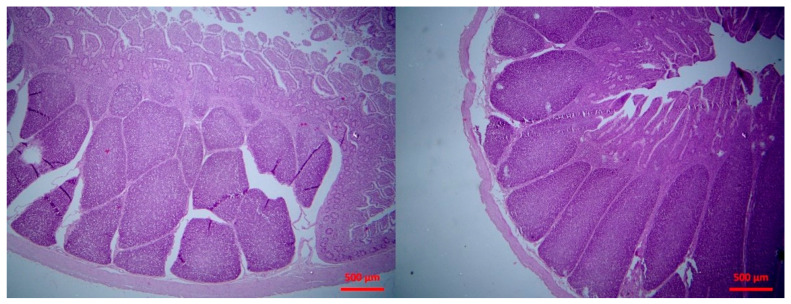
Small intestine mucosa: mild (**left** picture) to moderate (**right** picture) lymphofollicular hyperplasia (H&E, bar 500 μm).

**Figure 6:**
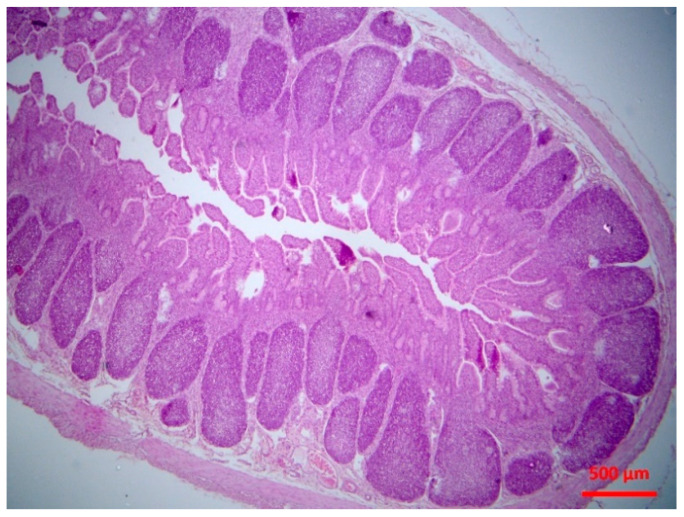
Small intestine mucosa: mild lymphofolicullar atrophy (H&E, bar 500 μm).

There was abomasitis with subepithelial presence of eosinophils, lymphocytes and plasma cells. This was consistently observed in all inoculated lambs, although there was a varying degree of the inflammation (mild to moderate) between animals. In the small intestine, there were various lesions when compared to healthy tissue (Figure 1). At the early stage of post-inoculation (up to D10), salient lesions included distension and oedema of intestinal villi (Figure 2), leucocytic infiltration (macrophages, neutrophils, lymphocytes, plasma cells) (Figure 3 and Figure 4) and hyperplasia of lymphoid nodules with apparent germinal centres (Figure 5). At later stages (D10 and thereafter), the lymphoid tissue was consistently observed to be atrophied and/or degenerated (Figure 6); there was also marked subepithelial infiltration of lymphocytes, plasma cells and eosinophils.

## 3. Discussion

After challenge, intestinal infection has been established in inoculated lambs. This was corroborated by the consistent isolation of the challenge organism from the lambs and the definite evidence of inflammation during the histopathological evaluation. Nevertheless, infection was mild and did not lead to fatalities as recorded with other *Salmonella* species [25]. The organism can nevertheless be confirmed as an intestinal pathogen. The recovery of an isolate from a field case of intestinal infection in a lamb, the subsequent use of this isolate in the experimental reproduction of a mild-type intestinal infection and the re-isolation of the organism from the experimental animals confirm the causality of the disease. The mild effects on the experimental animals should not deter from confirming this association, but rather they are indicative of a mild pathogenicity of the bacterium and effective defences of the animals.

The isolate caused subclinical damage which was evident soon after inoculation. There were clear pathological findings in the intestinal mucosa, as confirmed by the histopathological examination. A possible destruction of the villi (even of mild extent) can lead to problems of nutrient absorption, whilst the observed lymphofollicular atrophy may predispose to reduced intestinal defences. Whilst *S. enterica* subsp. *diarizonae* may be of low pathogenic significance on its own, it may pave a way for other bacteria to exert their pathogenicity in infected lambs, causing more significant gastrointestinal problems, which are of paramount importance in lambs [25].

At the end, the challenge isolate had disseminated outside the gastrointestinal tract and was isolated from liver and gallbladder tissue samples. The findings are in contrast to a hypothesis by Katribe et al. [26], who indicated that *S. enterica* subsp. *diarizonae* was limited in the intestinal tract. Many researchers believe that this specific subspecies may be a commensal resident of sheep’s intestinal tract [17]. However, our findings are allied more to the results of Lacasta et al. [27], who have also reported extra-intestinal (respiratory) infection of sheep with the pathogen. This shows the possibility for invasiveness of the organism; hence, in immunocompromised hosts, it might even be able to cause mortality. The results suggest that, whilst *S. enterica* subsp. *diarizonae* seems to be a host-adapted *Salmonella* subspecies, the organism can retain its pathogenic properties and, under certain conditions, may cause clinical conditions in sheep.

The isolation of the organism from buccal samples is of particular interest. Bacteria in the mouth of lambs could have originated from the inoculum or from regurgitation of gastric content; the latter possibility is more likely, as a gastric catheter was employed for inoculation, although the possibility of a leakage during the inoculation process cannot be ruled out. Consequentially, the isolation of *Salmonella* from milk samples of a ewe is consistent with the presence of the organism within the mouth of her offspring, whence it was likely transferred during sucking by lambs. We postulate that as the lower part of the teat comes into contact with the pharynx of the lamb [28], the organism was attached thereon, subsequently entering into the duct; perhaps the tongue of the lamb might have “pushed” the bacteria upwards into the duct. In previous studies [29], we have presented evidence that bacteria can be transferred from the mouth of lambs to the teat duct of their dams even after a short (1 min) sucking activity.

In a field study previously reported [20], *S. enterica* subsp. *diarizonae* was isolated from faecal samples of a lamb with grave clinical signs, as well as from faecal samples of a ewe. The present findings are in contrast to those of the field study. These contrasting findings can be attributed to a possibly reduced immune state of those animals, given that the field work was undertaken in a sheep flock a while earlier affected with bluetongue, which may cause immunosuppression in affected animals [30,31]. The finding of less frequent recoveries of the organism by microbiological techniques than the frequency of detection of nucleic acid by molecular techniques lends some support to this hypothesis. Likely, in the experimental study, effective defences of the host eliminated the challenged organism, despite the high dose administered. This is further supportive of a mild pathogenicity of the organism, as postulated above.

Detection of the *invA* gene was used to confirm the presence of *Salmonella* using conventional PCR. Although this gene is not specific in *S. enterica* subsp. *diarizonae*, it was selected because of its increased sensitivity for *Salmonella* detection [32]. Each sample that yielded the expected PCR product was interpreted to have harboured *S. enterica* subsp. *diarizonae*. The simultaneous presence of *Salmonella* strains beyond the challenge organism was considered unlikely to have occurred, especially with the findings of the PCR allied to the bacteriological results. In particular, the following points were taken into account: (a) no *Salmonella* was recovered from the animals before challenge, nor was the *invA* gene detected in any sample before challenge, (b) the experimental animals remained isolated from each other throughout the study, with limited and strictly controlled access to their pens, (c) *Salmonella* recovered from the samples after challenge was speciated as *S. enterica* subsp. *diarizonae*, (d) typing of the isolates from the experimental animals after challenge in all cases confirmed the identity of the isolates as *S. enterica* subsp. *diarizonae*.

The infection in sheep poses a zoonotic threat. Lamb consumption may contribute to potential human infections, as shown by the isolation of the organism from abattoir samples at a small but existent rate [14,15,16]. Moreover, the recovery of this organism from milk samples (present results) should increase awareness about the presence of previously unrecognised pathogens in the milk of ewes, which may subsequently be transferred to humans in cases of inappropriate thermal processing of milk. The detection of multi-resistant [33] and of colistin-resistant [15] isolates of the organism from animals should also be taken into account, when assessing the significance of the potential zoonotic risk of the organism.

## 4. Materials and Methods

### 4.1. Animals

In total, 16 clinically healthy lambs from 8 ewes were enrolled into the study on the first day of life. Of these, 12 lambs were challenged with *S. enterica* subsp. *diarizonae* serovar 61:k:1,5,(7). The dams of the lambs had been housed throughout their gestation and were provided with a commercial concentrate feed plus hay and barlay straw. During the final month of gestation and after lambing, ewes were housed individually; during the latter period, lambs were also penned with their respective dam.

Two examinations of blood samples for concentrations of β-hydroxybutyrate [34] did not reveal any problems: in all animals, concentrations were always below 0.95 mmol L^−1^. Further, examination of serum blood samples, by using ELISA tests with commercially available kits, for presence of antibodies against *Mycobacterium avium* subsp. *paratuberculosis* (ID Screen^®^ Paratuberculosis Indirect; ID VET, Grabels, France), *Small Ruminant Lentivirus* (ID Screen^®^ MVV / CAEV Indirect; ID VET) and *Bluetongue Virus* (ID Screen^®^ Bluetongue Competition, ID VET) [35,36,37], also did not reveal any problems: in no ewe, antibodies of the above pathogens were detected by any of these tests. Finally, bacteriological examination of faecal samples of the ewes performed during the final week of gestation performed by standard techniques (including the ISO 6579-1:2017 for detection of *Salmonella* [38]), did not reveal a *Salmonella* infection in these animals.

The experiment was performed under a licence issued by the Veterinary Authority of the Region of Thessaly (licence no. 1997/30.01.2019), which was the competent authority to allow and monitor the experimentation. Conditions prescribed by legislation of the European Union in relation to animal experimentation procedures (Council Directive 86/809/EEC) were met during this work.

### 4.2. Inoculation Procedure

The lambs were challenged with a *S. enterica* subsp. *diarizonae* serovar 61:k:1,5,(7) that had been isolated during the investigation of the outbreak of diarrhoeic syndrome in a sheep farm [20]. Inoculation of lambs was performed on the 1st day of life (D0).

For inoculation, the challenge isolate was cultured in brain heart infusion broth (Thermo Fisher Scientific-Oxoid, Waltham, MA, USA) for 12 h at 37 °C. The culture was centrifuged and the sediment diluted into 20 mL phosphate-buffer saline pH 7.3 (PBS). A quantity of 10 mL of PBS was aspirated with a sterile syringe and, through use of a sterile plastic gastric catheter, was slowly introduced into the abomasum of the newborn lambs, by following the standard principles of administration of oral solutions to lambs [26]. Lambs were maintained at that position for 3 min after end of the procedure. The inoculum varied from 0.75 × 10^9^ to 1.80 × 10^9^ colony-forming units, as estimated by the method of Miles and Misra [39].

For this method, 1 mL of the inoculum was diluted into 9 mL of sterile phosphate-buffer-saline (PBS) and then serial dilutions of the suspension were performed in sterile PBS (1 mL into 9 mL). A drop of 0.02 mL of each serial dilution was plated and spread onto a plate with Plate Count Agar (PCA) (Thermo Fisher Scientific-Oxoid) and allowed to stand for 20 min before aerobic incubation at 37 °C for 18 to 24 h. The dilutions were performed in triplicate and for each dilution, three plates were inoculated. Then, colony counts on plates were made in drop areas with 20 to 100 colonies. The results of the three plates from each series of dilutions were averaged; then, the three means were again averaged for the final result of the content of the inoculum.

Four lambs received 10 mL of sterile PBS by using the above technique and were used as uninfected controls.

### 4.3. Examination of Animals-Samplings

#### 4.3.1. Lambs

On D0, but before challenge, a detailed clinical examination was carried out in lambs. Blood samples were collected from the jugular vein for haematological examination. Faecal and buccal swab samples were collected for bacteriological examination for detection of *Salmonella* spp.

Post-challenge, clinical examinations and sample collection as above were performed at 6 h (D0 + 6 h), 12 h (D0 + 12 h), 1 day (D1) and 2 (D2), 4 (D4), 7 (D7), 10 (D10), 14 (D14) and 21 (D21) days. On D1 and thereafter, lambs were euthanised (*n* = 1 on each of D1 and D2, *n* = 2 on each of D4, D7, D10, D14, D21). The uninfected controls were euthanised on D4 (*n* = 1), D10 (*n* = 1) and D21 (*n* = 2).

A detailed post-mortem examination was performed in all euthanised lambs. Tissue samples from the abomasum, the small intestine, the liver and the gallbladder were collected for bacteriological examination by using standard techniques and for detection of *Salmonella* spp. DNA by using a conventional PCR assay. Further, tissue samples from the abomasum, the small intestine and the mesenteric lymph nodes were collected for histopathological examination.

#### 4.3.2. Ewes

On the same occasions as above, faecal swab and milk samples were collected from the ewes for bacteriological examination. Milk samples were collected aseptically, separately from each of the two mammary glands of each ewe [40].

### 4.4. Laboratory Examinations

#### 4.4.1. Haematological Examination

Samples for haematological examination were mixed by gentle repeated inversions for several seconds to avoid coagulation. They were processed within 30 min after collection. Initially, blood smears were prepared and kept dry at room temperature. A complete blood count was performed by an automated haematological analyser (Abbott Cell-Dyn 3500 System; Abbott, Abbott Park, IL, USA) previously evaluated in ovine haematology [41]. The following parameters were determined: haematocrit, erythrocyte count, haemoglobin concentration, mean corpuscular volume, mean corpuscular haemoglobin concentration, total leucocyte count and thrombocyte count. Blood smears were evaluated for leucocyte type differentiation and detection of potential presence of morphological abnormalities.

#### 4.4.2. Bacteriological Examination

Faecal swab samples from lambs and ewes were processed for isolation of *Salmonella*, after immersion into 1 mL of buffered peptone water. Buccal swab samples from lambs were processed similarly. For milk samples from ewes, a volume of 1 mL of milk was mixed with 10 mL of buffered peptone water.

Tissue samples from lambs collected during post-mortem examination were washed with PBS and were then homogenised (10 g of tissue sample with 50 mL of sterile PBS blended for 3 min) in a tissue blender (Mixwel; Alliance Bio Expertise, Guipry, France). Then, of the resulting fluid, 20 mL were added into 200 mL of buffered peptone water, which was followed by the same procedure as above.

After initial processing as above, the enriched suspensions were processed according to the ISO 6579-1:2017 for detection of *Salmonella* [38]; modified semi-solid Rappaport Vassiliadis medium (Thermo Scientific-Oxoid), XLD (Thermo Scientific-Oxoid) and Salmonella-Shigella agar (Thermo Scientific-Oxoid) agar were used. Colonies obtained were cultured onto sheep blood agar plates and McConkey plates for incubation at 37 °C for up to 48 h. Colonies grown were processed for identification by using the API rapid identification system (Biomerieux, Marcy-l’-Etoile, France). Then, the automated identification Vitek 2 system (Biomerieux) was employed for confirmation of identification of all *Salmonella* isolates obtained as above. Finally, eight isolates selected at random among the isolates recovered from all clinical samples (3 from faecal, 3 from buccal and 2 from milk samples), were subcultured and sent to the Greek National Reference Laboratory for Salmonellae (in animals), which is a service of the Greek Ministry of Rural Development and Food, for serotyping.

#### 4.4.3. Molecular Examination for Presence of Salmonella spp.

##### DNA Extraction

Faecal swab and buccal swab samples were washed with 0.2 mL and 0.4 mL, respectively, of PBS into a DNA-free 2 mL Eppendorf tube. For DNA extraction of milk samples, an initial volume of 1 mL was centrifuged for 10 min at 10,000× *g*; subsequently, the upper fatty layer was appropriately removed and the supernatant was discarded; the pellet that remained was used as the starting sample material. With regard to tissue samples, 0.05 g of tissue was placed into an Eppendorf tube with 0.5 mL of PBS and subjected to mixing in Vortex equipment (Velp Scientifica, Usmate, Italy) for 3 min; then, 0.5 mL of the mixture was transferred to a new Eppendorf tube.

Thereafter, in all the above, the DNA extraction procedure was carried out using PureLink^®^ Genomic DNA Kit K 1820-01; (Life Technologies, Karlsbad, CA, USA), according to the manufacturer’s instructions. The bound DNA was eluted using 0.1 mL elution buffer, split in aliquots of 5 μL and stored at −20 °C.

##### PCR Amplification

Presence of *Salmonella* spp. *invA* gene was detected by simple PCR assay. Details of primers and conditions employed are shown in Table 8. Amplification was performed in a PT-100 Thermocycler (MJ Research Inc., St Bruno, QC, Canada). Reactions were performed in a total volume of 50 μL PCR mixture, containing 0.045 mL of Platinum PCR SuperMix (Applied Biosystems, Foster City, CA, USA) and approximately 150 ng of the extracted DNA. The thermal cycling procedure consisted of a pre-denaturation step at 95 °C for 2 min, 35 cycles of denaturation at 94 °C for 1 min, annealing for 1 min and extending at 72 °C for 45 s and a final elongation step at 72 °C for 7 min. Subsequently, 0.005 mL of each product was analysed by electrophoresis on 1.5% agarose gel stained with ethidium bromide (100 mL 1× TBE buffer; DGel Electrosystem, Montreal, QC, Canada), 2 g agarose (NIPPON Genetics, Tokyo, Japan), 0.005 mL ethidium bromide (Sigma-Aldrich, Saint Louis, MO, USA) and observed at ultraviolet light. Each product equal in size to the expected amplicon was considered as positive.

#### 4.4.4. Histopathological Examination

Tissue samples were fixed in 10% neutral-buffered formalin and embedded in paraffin wax. Haematoxylin and eosin (H&E) standard staining procedures were performed for histopathological studies.

### 4.5. Data Management and Analysis

All data were entered into Microsoft Excel and analysed using IBM SPSS Statistics (ver. 21) (IBM; Armonk, NY, USA).

For estimation of incidence rates, we took into account that a lamb might change from not being infected to being infected and vice-versa; during the interval between sampling points, it was not possible to know what had happened between the two sampling points, i.e., how many cases of infection and “cures” might have occurred. The model detailed by Mavrogianni et al. [43] was used and appropriately modified for the tissues under evaluation in the present study. Based on the above, it was possible to calculate incidence rates of the various infections. Further, it was possible to estimate the length of time for which an animal was at risk before it became infected, as well as the length of time that an animal had been infected. Incidence rate was defined as the proportion of animals at risk, which developed the condition when the time at risk was the same in each group.

Linear mixed models were used in analysis to account for repeated measures of values of haematological parametres over the course of the study. Time points of collecting data were selected as within-subject variables and group allocation as a between-subject factor. Independent variables (fixed effects) included study group, sampling point and a sampling point–study group interaction.

The various associations were evaluated in a table of cross-categorised frequency data by use of the Pearson chi-square test or the Fisher exact test as appropriate.

In all cases, statistical significance was defined as *p* < 0.05.

## 5. Conclusions

The general conclusion from the study can be that *S. enterica* subsp. *diarizonae* is an opportunistic gastrointestinal pathogen in lambs. The organism can cause a mild infection, with faecal shedding and definite histopathological changes in the gastrointestinal tract; the bacteria can be isolated from the internal organs of infected animals. The organism can be transmitted from lambs to ewes during sucking, with potential for subsequent isolation of the organism from the milk of the dams of infected lambs.

## Figures and Tables

**Table 1:** Haematological findings (median values) in lambs orally inoculated with *S. enterica* subsp. *diarizonae*.

	**Haematocrit (%)**		**Erythrocytes (×10^6^ cells μL^−1^)**		**Haemoglobin (g dL^−1^)**		**MCV (fL) ^1^**		**MCHC (g dL^−1^) ^1^**	
Sampling occasion	Inoculated lambs	Uninfected controls	Inoculated lambs	Uninfected controls	Inoculated lambs	Uninfected controls	Inoculated lambs	Uninfected controls	Inoculated lambs	Uninfected controls
D0	33.8	26.1	8.2	10.2	10.5	11.1	40.4	45.2	12.8	12.4
D0 + 6 h	30.6	28.0	7.6	10.4	9.8	10.7	39.7	44.6	12.7	12.7
D0 + 12 h	28.9	28.1	7.3	11.1	9.5	10.9	39.0	44.2	12.7	12.9
D1	30.0	28.3	7.7	11.4	9.4	11.1	39.2	45.0	12.4	12.4
D2	30.4	27.7	7.7	10.7	9.7	10.6	39.6	44.2	12.7	11.0
D4	28.1	28.9	7.2	11.0	9.0	11.4	38.7	41.2	12.3	11.0
D7	28.9	29.1	7.7	11.9	9.1	12.0	35.1	41.7	11.9	11.6
D10	28.6	29.2	8.1	9.8	8.8	11.4	35.2	40.4	10.9	10.5
D14	27.8	28.3	8.7	9.9	8.3	10.5	32.7	40.7	9.8	9.6
D21	34.4	28.7	10.5	10.1	10.3	10.5	33.2	41.3	10.0	10.3
	**Total leucocytes (cells μL^−1^)**		**Neutrophils (cells μL^−1^)**		**Neutrophils (% leucocytes)**		**Lymphocytes (cells μL^−1^)**		**Lymphocytes (% leucocytes)**	
Sampling occasion	Inoculated lambs	Uninfected controls	Inoculated lambs	Uninfected controls	Inoculated lambs	Uninfected controls	Inoculated lambs	Uninfected controls	Inoculated lambs	Uninfected controls
D0	4740	6425	2100	3300	42.1	51.0	2610	2620	49.7	40.9
D0 + 6 h	4650	7010	1945	4040	37.9	57.6	2815	2540	55.3	36.2
D0 + 12 h	4720	8200	1460	5280	29.1	64.4	2720	2650	63.2	32.3
D1	5270	5350	1550	3400	31.8	63.5	3330	1730	63.1	32.3
D2	5300	5470	1790	1800	32.2	40.6	3440	2215	60.4	51.2
D4	4970	5000	1480	730	29.5	14.6	3050	2000	60.8	80.0
D7	4935	5085	1230	2255	27.2	44.4	3475	2555	66.1	50.0
D10	5395	4650	990	1845	17.9	40.0	4025	2520	74.8	53.6
D14	7280	4105	2330	1580	27.5	38.2	5260	2290	63.8	56.2
D21	10,725	9490	3475	3250	34.3	34.4	5675	2325	53.0	55.8
	**Monocytes (cells μL^−1^)**		**Monocytes (% leucocytes)**		**Eosinophils (cells μL^−1^)**		**Eosinophils (% leucocytes)**			
Sampling occasion	Inoculated lambs	Uninfected controls	Inoculated lambs	Uninfected controls	Inoculated lambs	Uninfected controls	Inoculated lambs	Uninfected controls		
D0	155	65	2.6	0.9	50	285	1.0	4.6		
D0 + 6 h	120	20	3.2	0.3	70	260	1.3	3.7		
D0 + 12 h	60	20	1.6	0.2	40	110	1.0	1.3		
D1	60	20	1.5	0.4	60	70	1.5	1.2		
D2	245	180	4.0	4.0	40	45	0.7	1.0		
D4	80	20	1.6	0.4	50	40	1.0	0.8		
D7	70	100	1.3	2.0	50	45	0.9	0.9		
D10	115	100	1.8	2.2	35	85	0.8	2.1		
D14	190	50	2.1	1.2	130	120	1.7	2.2		
D21	150	105	1.4	1.1	1140	600	9.1	6.4		
	**Basophils (cells μL^−1^)**		**Basophils (% leucocytes)**		**Thrombocytes (cells μL^−1^)**					
Sampling occasion	Inoculated lambs	Uninfected controls	Inoculated lambs	Uninfected controls	Inoculated lambs	Uninfected controls				
D0	90	110	1.9	1.7	545	763				
D0 + 6 h	120	100	2.1	1.4	556	893				
D0 + 12 h	100	90	2.0	1.0	512	844				
D1	90	80	2.0	1.5	549	922				
D2	90	70	1.9	1.7	598	1030				
D4	110	180	2.2	3.6	934	1045				
D7	120	95	2.6	1.8	1028	1770				
D10	95	55	1.7	1.2	1146	1503				
D14	90	45	1.2	1.1	1151	1167				
D21	185	100	1.2	1.0	1082	1070				

^1^ MCV: mean corpuscular volume, MCHC: mean corpuscular haemoglobin concentration.

**Table 2:** Bacteriological findings (isolation of *Salmonella*) in faecal or buccal swab samples from lambs orally inoculated with *S. enterica* subsp. *diarizonae* (results expressed as positive of total samples examined).

Before Challenge	Day After Challenge									Cumulative
D0	D0 + 6 h	D0 + 12 h	D1	D2	D4	D7	D10	D14	D21	
Faecal samples										
0/12	8/12	12/12	11/12	9/11	2/10	2/8	1/6	0/4	0/2	45/77
Buccal samples										
0/12	0/12	0/12	6/12	2/11	1/10	1/8	0/6	0/4	0/2	10/77

**Table 3:** Bacteriological findings (isolation of *Salmonella*) in tissue samples from lambs orally inoculated with *S. enterica* subsp. *diarizonae* and euthanised (results expressed as positive of total samples examined).

Tissue	Day After Challenge							Cumulative
	D1	D2	D4	D7	D10	D14	D21	
small intestine	1/1	1/1	0/2	1/2	1/2	2/2	0/2	6/12
abomasum	1/1	1/1	2/2	0/2	0/2	0/2	0/2	4/12
liver	0/1	0/1	1/2	0/2	0/2	1/2	1/2	3/12
gallbladder	0/1	0/1	1/2	1/2	0/2	0/2	0/2	2/12
Cumulative	2/4	2/4	4/8	2/8	1/8	3/8	1/8	15/48

**Table 4:** Association between concurrent isolation of *Salmonella* from faecal swab samples and from tissue samples from the same animals from lambs orally inoculated with *S. enterica* subsp. *diarizonae* and euthanised (results expressed as number of lambs with samples in each category).

		Recovery from Faecal Swab Samples	
		Yes	No
**Recovery from Small Intestine Tissue Samples**	Yes	2	4
	No	0	6
**Recovery from Any Tissue Samples**	Yes	3	8
	No	1	0

**Table 5:** Detection of *invA* gene in faecal or buccal swab samples from lambs orally inoculated with *S. enterica* subsp. *diarizonae* (results expressed as positive of total samples examined).

Before Challenge	Day After Challenge									Cumulative
D0	D0 + 6 h	D0 + 12 h	D1	D2	D4	D7	D10	D14	D21	
Faecal samples										
0/12	9/12	12/12	11/12	10/11	10/10	8/8	6/6	4/4	0/2	70/77
Buccal samples										
0/12	3/12	3/12	8/12	5/11	3/10	2/8	0/6	0/4	0/2	24/77

**Table 6:** Frequency of detection of the *invA* gene in various samples collected from lambs orally inoculated with *S. enterica* subsp. *diarizonae* and euthanised or from their dams (results expressed as positive of total examined).

Animals	Type of Samples	No. of Animals in Samples from which Detected	No. of Samples in which Detected
Lambs	Faeces	12/12 (1.000)	70/77 (0.909)
Lambs	Buccal cavity swab	9/12 (0.750)	24/77 (0.312)
Ewes	Faeces	0/6 (0.000)	0/39 (0.000)
Ewes	Milk	2/6 (0.333)	3/78 (0.038) ^1^
Lambs	Small intestine tissue	12/12 (1.000)	12/12 (1.000)
Lambs	Abomasum tissue	8/12 (0.667)	8/12 (0.667)
Lambs	Liver tissue	12/12 (1.000)	12/12 (1.000)
Lambs	Gallbladder tissue	8/12 (0.667)	8/12 (0.667)

^1^ Corresponding to 3/39 sampling occasions (0.078).

**Table 7:** Detection of *invA* gene in tissue samples from lambs orally inoculated with *S. enterica* subsp. *diarizonae* and euthanised (results expressed as positive of total samples examined).

Tissue	Day After Challenge							Cumulative
	D1	D2	D4	D7	D10	D14	D21	
small intestine	1/1	1/1	2/2	2/2	2/2	2/2	2/2	12/12
abomasum	1/1	1/1	2/2	2/2	1/2	1/2	0/2	10/12
liver	1/1	1/1	2/2	2/2	2/2	2/2	2/2	12/12
gallbladder	0/1	0/1	1/2	1/2	2/2	2/2	2/2	8/12
Cumulative	3/4	3/4	7/8	7/8	7/8	7/8	6/8	15/48

**Table 8:** Primers used and work conditions undertaken for detection of *Salmonella* spp. *invA* gene in DNA extract from samples from lambs or ewes.

Primer Sequence	Concentration (μM)	Product Size (bp)	AT ^1^ (°C)	Reference
Fw-GTGAAATTATCGCCACGTTCGGGCAA	0.25	284	55.0	[42]
Rv-TCATCGCACCGTCAAAGGAACC				

^1^ AT: annealing temperature.

## Data Availability

All data for this work are within the manuscript and in the publicly available PhD thesis of the first author (www.didaktorika.gr).
